# Seroprevalence of SARS-CoV-2 antibodies in social housing areas in Denmark

**DOI:** 10.1186/s12879-022-07102-1

**Published:** 2022-02-10

**Authors:** Kamille Fogh, Alexandra R. R. Eriksen, Rasmus B. Hasselbalch, Emilie Sofie Kristensen, Henning Bundgaard, Susanne D. Nielsen, Charlotte S. Jørgensen, Bibi F. S. S. Scharff, Christian Erikstrup, Susanne G. Sækmose, Dorte K. Holm, Bitten Aagaard, Jakob Norsk, Pernille Brok Nielsen, Jonas H. Kristensen, Lars Østergaard, Svend Ellermann-Eriksen, Berit Andersen, Henrik Nielsen, Isik S. Johansen, Lothar Wiese, Lone Simonsen, Thea K. Fischer, Fredrik Folke, Freddy Lippert, Sisse R. Ostrowski, Steen Ethelberg, Anders Koch, Anne-Marie Vangsted, Tyra Grove Krause, Anders Fomsgaard, Claus Nielsen, Henrik Ullum, Robert Skov, Kasper Iversen

**Affiliations:** 1 Department of Cardiology, Copenhagen University Hospital, Herlev and Gentofte, Borgmester Ib Juuls Vej 1, 2730 Herlev, Denmark; 2 Department of Emergency Medicine, Copenhagen University Hospital, Herlev and Gentofte, Herlev, Denmark; 3 Department of Cardiology, Copenhagen University Hospital, Rigshospitalet, Copenhagen, Denmark; 4 Department of Infectious Diseases, Copenhagen University Hospital, Rigshospitalet, Copenhagen, Denmark; 5 Statens Serum Institut, Copenhagen, Denmark; 6 Department of Clinical Immunology, Copenhagen University Hospital, Rigshospitalet, Copenhagen, Denmark; 7 Department of Clinical Microbiology, Aarhus University Hospital, Aarhus, Denmark; 8 Department of Clinical Immunology, Zealand University Hospital, Køge, Denmark; 9 Department of Clinical Immunology, Odense University Hospital, Odense, Denmark; 10 Department of Clinical Immunology, Aalborg University Hospital, Aalborg, Denmark; 11 Department of Infectious Diseases, Aarhus University Hospital, Aarhus, Denmark; 12 University Research Clinic for Cancer Screening, Randers Regional Hospital, Randers, Denmark; 13 Department of Infectious Diseases, Aalborg University Hospital, Aalborg, Denmark; 14 Department of Infectious Diseases, Odense University Hospital, Odense, Denmark; 15 Department of Infectious Diseases, Zealand University Hospital, Roskilde, Denmark; 16 Department of Science and Environment, Roskilde University, Roskilde, Denmark; 17 Department of Clinical Research, North Zealand Hospital, Hillerød, Denmark; 18 Copenhagen Emergency Medical Services, Copenhagen, Denmark; 19 Department of Clinical Medicine, University of Copenhagen, Copenhagen, Denmark; 20 Department of Clinical Medicine, Aarhus University, Aarhus, Denmark; 21 Department of Clinical Research, University of Southern Denmark, Odense, Denmark; 22 Department of Clinical Medicine, Aalborg University, Odense, Denmark; 23 Department of Public Health, University of Copenhagen, Copenhagen, Denmark

**Keywords:** SARS-CoV-2, COVID-19, Seroprevalence, Social housing areas, Antibodies

## Abstract

**Background:**

COVID-19 is thought to be more prevalent among ethnic minorities and individuals with low socioeconomic status. We aimed to investigate the prevalence of SARS-CoV-2 antibodies during the COVID-19 pandemic among citizens 15 years or older in Denmark living in social housing (SH) areas.

**Methods:**

We conducted a study between January 8th and January 31st, 2021 with recruitment in 13 selected SH areas. Participants were offered a point-of-care rapid SARS-CoV-2 IgM and IgG antibody test and a questionnaire concerning risk factors associated with COVID-19. As a proxy for the general Danish population we accessed data on seroprevalence from Danish blood donors (total Ig ELISA assay) in same time period.

**Results:**

Of the 13,279 included participants, 2296 (17.3%) were seropositive (mean age 46.6 (SD 16.4) years, 54.2% female), which was 3 times higher than in the general Danish population (mean age 41.7 (SD 14.1) years, 48.5% female) in the same period (5.8%, risk ratios (RR) 2.96, 95% CI 2.78–3.16, p > 0.001). Seropositivity was higher among males (RR 1.1, 95% CI 1.05–1.22%, p = 0.001) and increased with age, with an OR seropositivity of 1.03 for each 10-year increase in age (95% CI 1.00–1.06, p = 0.031). Close contact with COVID-19-infected individuals was associated with a higher risk of infection, especially among household members (OR 5.0, 95% CI 4.1–6.2 p < 0,001). Living at least four people in a household significantly increased the OR of seropositivity (OR 1.3, 95% CI 1.0–1.6, p = 0.02) as did living in a multi-generational household (OR 1.3 per generation, 95% CI 1.1–1.6, p = 0.003). Only 1.6% of participants reported not following any of the national COVID-19 recommendations.

**Conclusions:**

Danish citizens living in SH areas of low socioeconomic status had a three times higher SARS-CoV-2 seroprevalence compared to the general Danish population. The seroprevalence was significantly higher in males and increased slightly with age. Living in multiple generations households or in households of more than four persons was a strong risk factor for being seropositive. Results of this study can be used for future consideration of the need for preventive measures in the populations living in SH areas.

**Supplementary Information:**

The online version contains supplementary material available at 10.1186/s12879-022-07102-1.

## Background

The first confirmed case of SARS-CoV-2 infection in Denmark was reported on February 27, 2020 and by May 4th, 2021 there have been more than 254,482 confirmed cases of SARS-CoV-2 infection and more than 2491 COVID-19 related deaths in Denmark [1]. The epidemic in Denmark was characterized by two infection waves in spring 2020 and autumn/winter 2021.

So far, the outbreak of the epidemic has had a heterogeneous regional patterns with geographical accumulations and varying incidence by gender, age, social class and employment [2]. Although there is equal and free of charge access to health care for everybody in Denmark including testing for COVID-19 (viral throat- and nasopharyngeal swab), citizens’ behavior may vary in different social segments. National and regional seroprevalence data offer valuable information to tailor public health policies towards the COVID-19 epidemic.

According to the Danish authorities, 15 residential areas are currently defined as social housing (SH) areas, characterized by low employment rates, low income, low education level, high crime rate and/or increased proportion of immigrants [3]. Some reports suggest that ethnic minorities in a number of countries are over-represented among those infected with COVID-19, just as socioeconomic inequality is described among SARS-CoV-2 infected persons [4–6]. A Danish report from October 2020 showed similar patterns, where people of non-Western background accounted for 25.7% of cases with SARS-CoV-2 infection, despite representing only 8.9% of the population [7, 8].

Vulnerable and marginalized populations, certain ethnic minorities and persons of low socioeconomic status may have difficulties receiving and following health recommendations [9]. Which could lead to reduced use of protective equipment and difficulties in navigating the health care system with impaired contact, due to cultural and linguistic barriers, with the risk of being underdiagnosed. For cultural and economic reasons, individuals in SH areas may live in crowded multi-generational households with children, parents and grandparents, which has been hypothesized to increase transmission of SARS-CoV-2 [4, 10]. This may not only affect their households but also people in their environment. Estimating the contributions of individual and sociocultural factors that may lead to COVID-19 outbreaks is important, and systematic screening for antibodies against SARS-CoV-2 is an important tool in the surveillance of the current pandemic.

The Danish prevalence of SARS-CoV-2 seropositivity is reported for blood donors [11], medical students [12] hospital staff [13] and in a random sample of Danish citizens [14], but not in a subpopulation that may be at increased risk of SARS-CoV-2 infection due to low socioeconomic status.

In this study we determined the prevalence of SARS-CoV-2 antibodies among Danish citizens in SH areas at national and regional levels, by the use of Point-of care rapid test (POCT) for antibodies against SARS-CoV-2 and explored possible risk factors of seropositivity.

## Methods

### Study design and participation

The sero-epidemiological survey of SARS-CoV-2 infection in Denmark “Testing Denmark” is a nationwide surveillance study to investigate seropositivity for SARS-CoV-2 in the Danish population throughout the country, launched in September 2020.

The prevalence of SARS-CoV-2 antibodies among Danish citizens in SH areas was assessed by use of POCT during the period January 8th and January 31st, 2021 as part of “Testing Denmark”. By January 7th, 2021 there had been a total of 176.837 (3.0%) inhabitants tested positive for SARS-CoV-2 by PCR in Denmark [15].

**Fig. 1:**
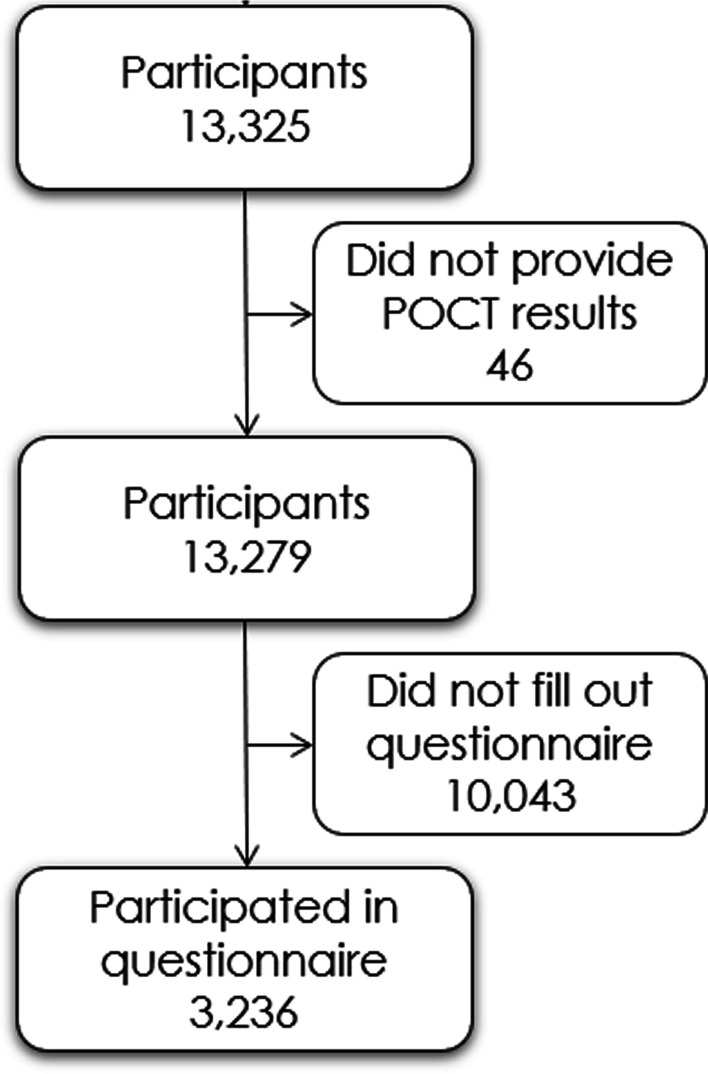
CONSORT diagram

Recruitment sites were chosen in collaboration with non-governmental organization with an ethnic minority background, who do voluntary efforts in their local community area. We recruited participants from 13 different SH areas in Denmark by convenience sampling, (see Additional file 1: Appendix, Fig. 1). All persons over 15 years of age living in the SH areas were invited to participate.

At all recruitment sites written information about the project were available in seven different languages; Danish, English, Arabic, Turkish, Farsi, Somali and Urdu.

### Questionnaire

Participants were asked to fill in a short questionnaire provided at the recruitment site, available in the seven different languages, due to risk factors, COVID-19 related symptoms, household, employment and behavior according to general recommendations from the Danish authorities (see Questionnaire in Additional file 1: Appendix).

Answers to the questionnaire and results of the POCT were managed in the Research Electronic Data Capture (REDCap), a secure web-based, electronic data capture tool, hosted at the Capital Region’s server [16, 17]. All personal data was kept in accordance with the general data protection regulation and data protection law stated by the Danish Data Protection Agency.

### Detection of SARS CoV-2 antibodies

The OnSite COVID-19 IgG/IgM Rapid Test (CTK Biotech inc., Poway, California, United States of America) is a single use lateral flow chromatographic immunoassay for qualitative detection and differentiation of IgG and IgM antibodies to SARS-CoV-2 in whole blood, which yields results in 15 min. This test was used by the participant with assistance from the project personnel, according to the manufacturer’s recommendations.

The manufacturer reported the POCT sensitivity and specificity of 96.9% (95% CI 96.7–98.5%) and 99.4% (95% CI 97.8–99.8%) respectively [18]. A comparative study (cases = 30 individuals, controls = 10 individuals) showed a slightly lower sensitivity of 90.0% and a specificity of 100% [19].

Fingerprick blood and detection buffer were added to the test cassette and test results were available after 15 min by trained project personnel. When no control line appeared or if the reading chamber was discolored by blood the test was inconclusive. Inconclusive test results were treated as negative. Weak signals for IgM and/or IgG, were considered positive. Participants were categorized as seropositive if they had developed IgG and/or IgM anti-SARS-CoV-2 antibodies.

### SARS-CoV-2 antibody levels in the general population

Since October 2020, all Danish blood donations are routinely screened for SARS-CoV-2 antibodies using the Wantai SARS-CoV-2 Ab ELISA (Beijing Wantai Biological Pharmacy Enterprise, Beijing, China). A comparative study with 129 non-hospitalized versus 31 hospitalized individuals diagnosed with SARS-CoV-2 showed a sensitivity of 96.7 (95% CI 92.4–98.6) and a specificity of 99.5 (95% CI 98.7–99.8) of the Wantai assay [20]. In this study we used anonymized data from January 2021, matched by period. This group was used as a proxy for the general population.

### Primary outcome

The primary outcome was the proportion of the study population with a positive antibody test for SARS-CoV-2 stratified by place of testing compared to the general population.

### Statistical analysis

Baseline characteristics and exposures are presented as n (%) for factors and mean (standard deviation (SD) or median (interquartile range (IQR)) for numeric variables as appropriate, and tested using either Students t-test or Chi square test. Household size was presented both as the total number of persons (with a maximum of > 5 according to the questionnaire) and as the number of generations in the household. The three generations were defined as individuals per household; < 19, 19–64 and > 65 years of age.

Unadjusted risk was presented as risk ratios (RR) with 95% confidence intervals (95% CI). To account for the possible clustering effect of participants living close to each other, we chose to use logistic regression, adjusting for test location (SH area) to determine the correlation between putative risk factors including age, gender, BMI, smoking, alcohol use, drug use, working, working in healthcare or nursing, household size, different exposures to SARS-CoV-2 in your social circle, and seropositivity. Adjusted analyses included age, gender and SH area. Results of these regression analyses were reported as odds ratios (OR) of risk factors and presented with 95% confidence intervals (95% CI). A p-value of < 0.05 was considered significant. Calculation were done using R version 6.3.1 [21]. A map of seropositivity were made using ggplot2 from tidyverse and in an interactive version using the tmap package in Rstudio.

## Results

### Characteristics

Between January 8th and January 31st, 2021, we included a total of 13,279 participants in SH areas. The mean age of the cohort was 46.6 (SD 16.4) years and 54.2% were female. Baseline characteristics of the cohort is shown in Table 1. A total of 3236 (24.4%) completed the accompanying questionnaire, primarily in Danish (94.4%). A flowchart of participant inclusion is depicted in Fig. 1.

**Table 1:** Baseline characteristics of the study cohort of people in SH areas stratified by seropositivity

Full cohort	Seronegative	Seropositive	p
n	10,983	2296	
Age (mean (SD))	46.43 (16.4)	47.27 (16.0)	0.031
Female (%)	5567 (54.8)	1100 (51.0)	0.001
Questionnaire cohort			
n	2632	604	
Body mass index (median [IQR])	25.35 [22.83, 29.03]	25.47 [23.12, 29.05]	0.476
Ever smoker (%)	727 (27.8)	121 (20.1)	< 0.001
Alcohol intake (%)	2075 (80.3)	458 (76.5)	0.039
Alcohol use (%)	140 (5.5)	21 (3.6)	0.074
Ever used drugs (%)	123 (4.7)	12 (2.0)	0.004
Education level (%)			0.842
No formal education	66 (2.5)	16 (2.7)	
Primary education	251 (9.6)	61 (10.2)	
Secondary education (youth education)	267 (10.3)	66 (11.0)	
Vocational training or short-term/medium-term higher education	1,384 (53.2)	325 (54.3)	
Long-term higher education	605 (23.2)	126 (21.0)	
Unknown	30 (1.2)	5 (0.8)	

Alcohol intake: Intake of alcohol within the past 12 months

Alcohol use: Reporting > 7 units of alcohol a week for females or > 14 units of alcohol for males

Participants were recruited from 13 selected SH areas placed in the municipalities of Copenhagen (n = 5816), Aarhus (n = 3718), Odense (n = 1731), Hoeje Taastrup (n = 1308), Helsingoer (n = 405) and Slagelse (n = 301), illustrated in Additional file 2: Table S1 and Additional file 1: Figure S1.

### Seroprevalence

A total of 2296 (17.3%) participants were seropositive for SARS-CoV-2 antibodies (IgG and/or IgM), of whom 1594 (12.0%) were positive for IgG antibodies, 1602 (12.1%) were positive for IgM antibodies and 899 (6.77%) were positive for both IgG and IgM antibodies. This was significantly higher than the seropositivity of 5.8% of the general population (n = 22,677, age 41.7 (SD 14.1) years, 48.5% female). Additional file 1: Figure S1 illustrates the seroprevalence in the general population and our study group. The RR range between SH areas varied from 1.1 in Ringerparken in Slagelse to 4.0 in Vollsmose in Odense.

**Fig. 2:**
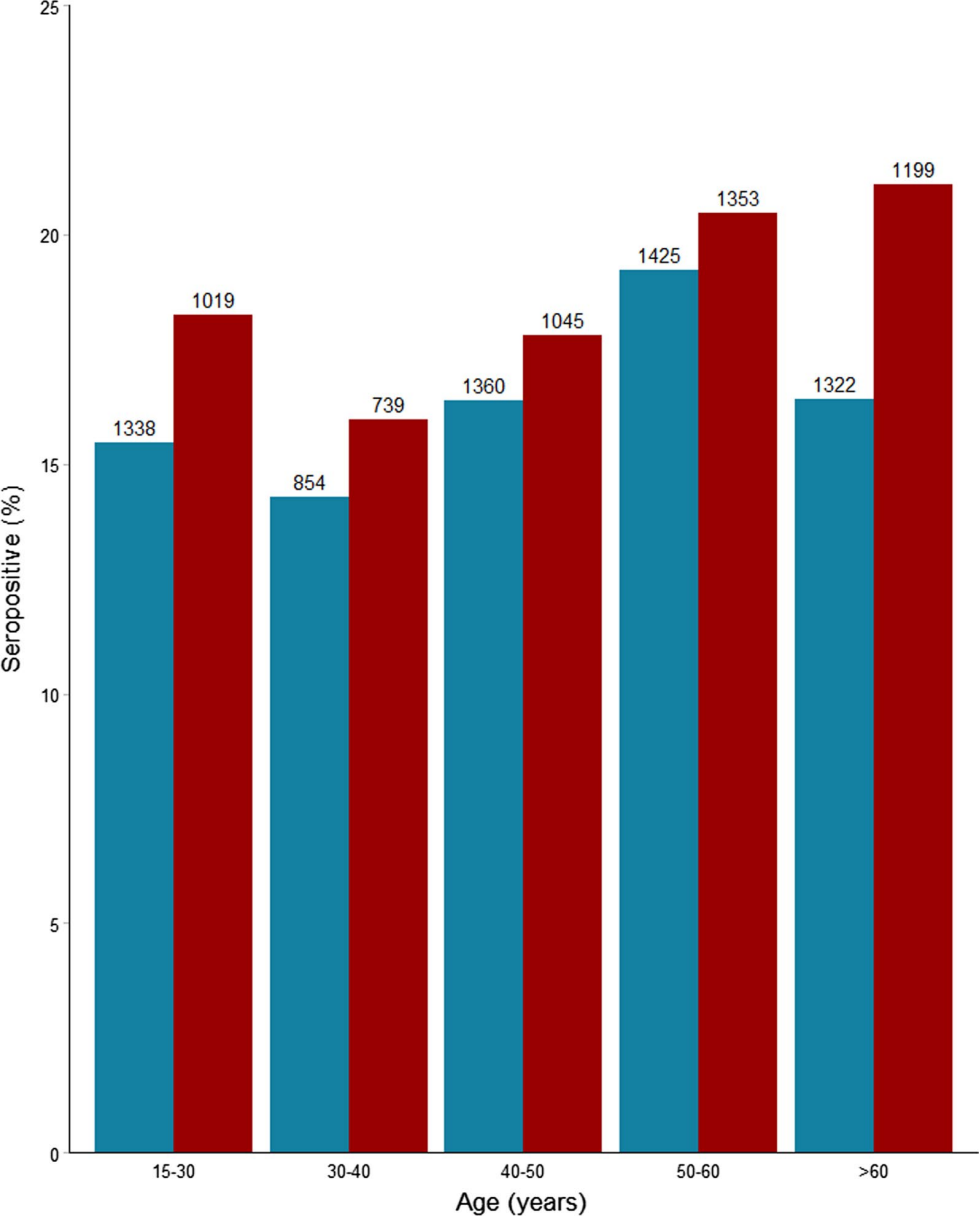
SARS-CoV-2 seroprevalence among 11,654 individuals in SH areas by age and sex. Red: male, blue: female, number of participants in each group

Seropositive participants were older than seronegative participants (47.3 vs 46.4 years, p = 0.03) and more likely to be male (RR 1.1, 95% CI 1.05–1.22%, p = 0.001). Seropositivity stratified on age and gender is shown in Fig. 2. Both age and gender remained associated with seropositivity in the multiple logistic regression model for gender and place of testing, for an increase in age of 10 years, OR of seropositivity was 1.03 (95% CI 1.00–1.06, p = 0.03) and for male gender OR 1.17 (95% CI 1.07–1.29, p = 0.001).

### Risk factors of infection

Seropositivity stratified by risk factors are shown in Table 2. In the analysis adjusted for test location several factors were associated with an increased odds of infection. Reporting a stay of > 15 min in a room with an infected person (OR 2.7, 95% CI 2.2–3.5, p < 0.001), having someone infected in the household (OR 5.0, 95% CI 4.1–6.2, p < 0.001), having bodily contact with someone infected (OR 1.2, 95% CI 1.0–1.5, p = 0.03) and having a family member with COVID-19 (OR 1.2, 95% CI 1.0–1.5, p = 0.03) were all associated with higher odds of infection. Seropositive participants were less likely to smoke (p < 0.001), consume alcohol (p = 0.04) and engage in drug use (p = 0.004) (Table 1). In multivariate logistic regression adjusted for age, gender and place of testing, smoking (OR 0.61, 95% CI 0.48–0.77, p < 0.001) and drug use (OR 0.44, CI 0.13–0.26, p = 0.01) were both significantly protective factors.

**Table 2:** Risk factors stratified by seropositivity of the questionnaire cohort of people in SH areas

	Seronegative	Seropositive	p
n	2632	604	
Stayed in the same room for 15 min with COVID-19 infected (%)	807 (46.7)	281 (70.8)	< 0.001
Had bodily contact with COVID-19 infected person (%)	415 (20.0)	180 (39.0)	< 0.001
Had worked/studied with COVID-19 infected person (%)	777 (35.1)	171 (36.5)	0.584
Had someone in the household infected with COVID-19 (%)	296 (12.2)	228 (41.3)	< 0.001
Had someone in the family or friend outside household infected with COVID-19 (%)	1445 (59.0)	352 (64.5)	0.021

**Fig. 3:**
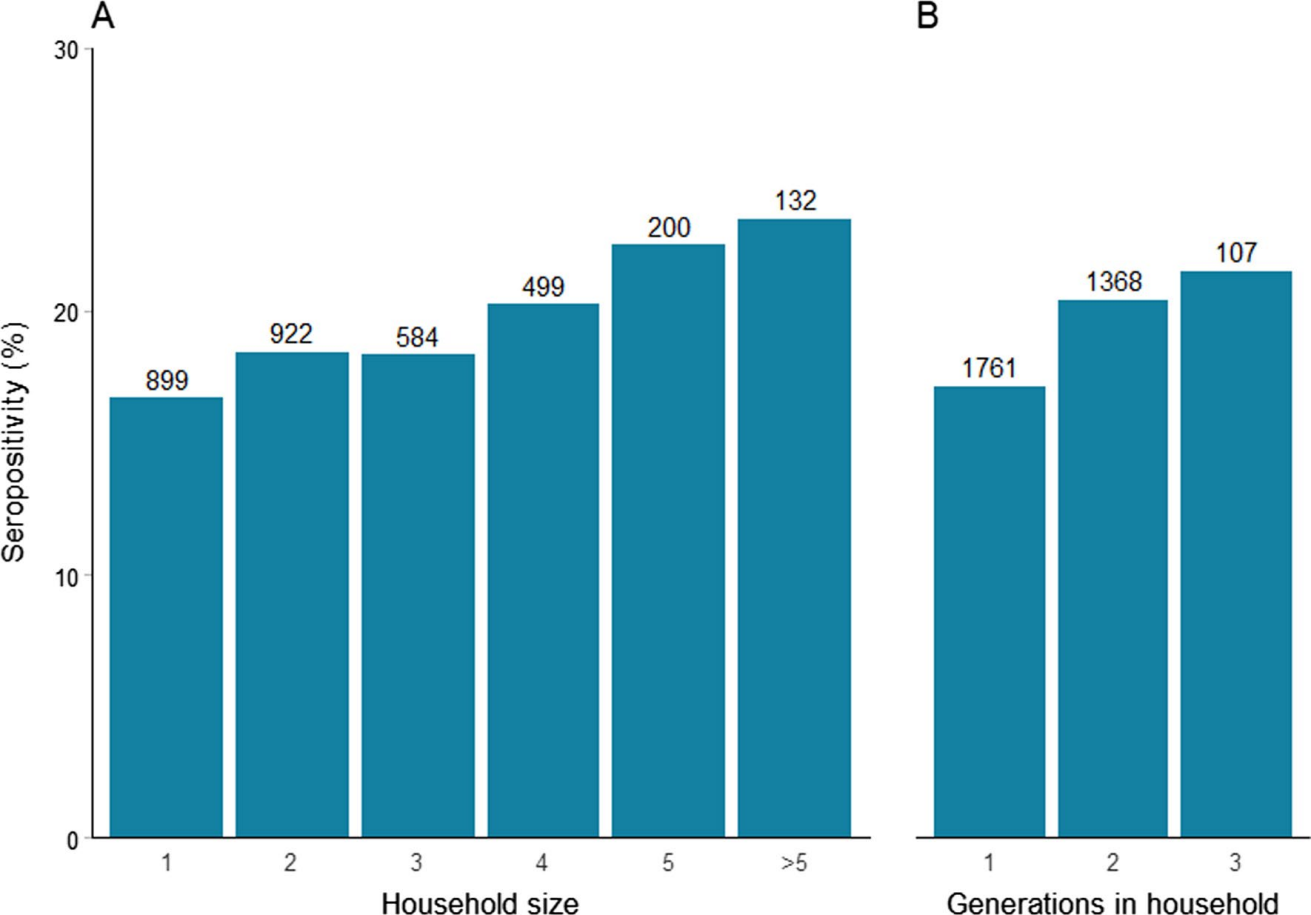
SARS-CoV-2 seroprevalence among 3236 individuals in SH areas by household size and generations in households. **A** Number of household members, **B** household size in terms of generations

Figure 3 shows the risk of seropositivity stratified by the size of the participants household. There was a clear association between the size of households and seropositivity. In adjusted analysis living at least four people in a household was associated with a significantly increased risk of seropositivity (OR 1.3, 95% CI 1.0–1.6, p = 0.02). Seroprevalence among participants living in a household with only one generation was 17.1%, with two generations 20.4% and with three or more generations 21.5%. Adjusted for age, gender and place of testing, living at least two generations in a household was associated with a significantly increased risk of seropositivity (OR 1.3, 95% CI 1.1–1.6, p = 0.003) as compared to living only one generation.

**Fig. 4:**
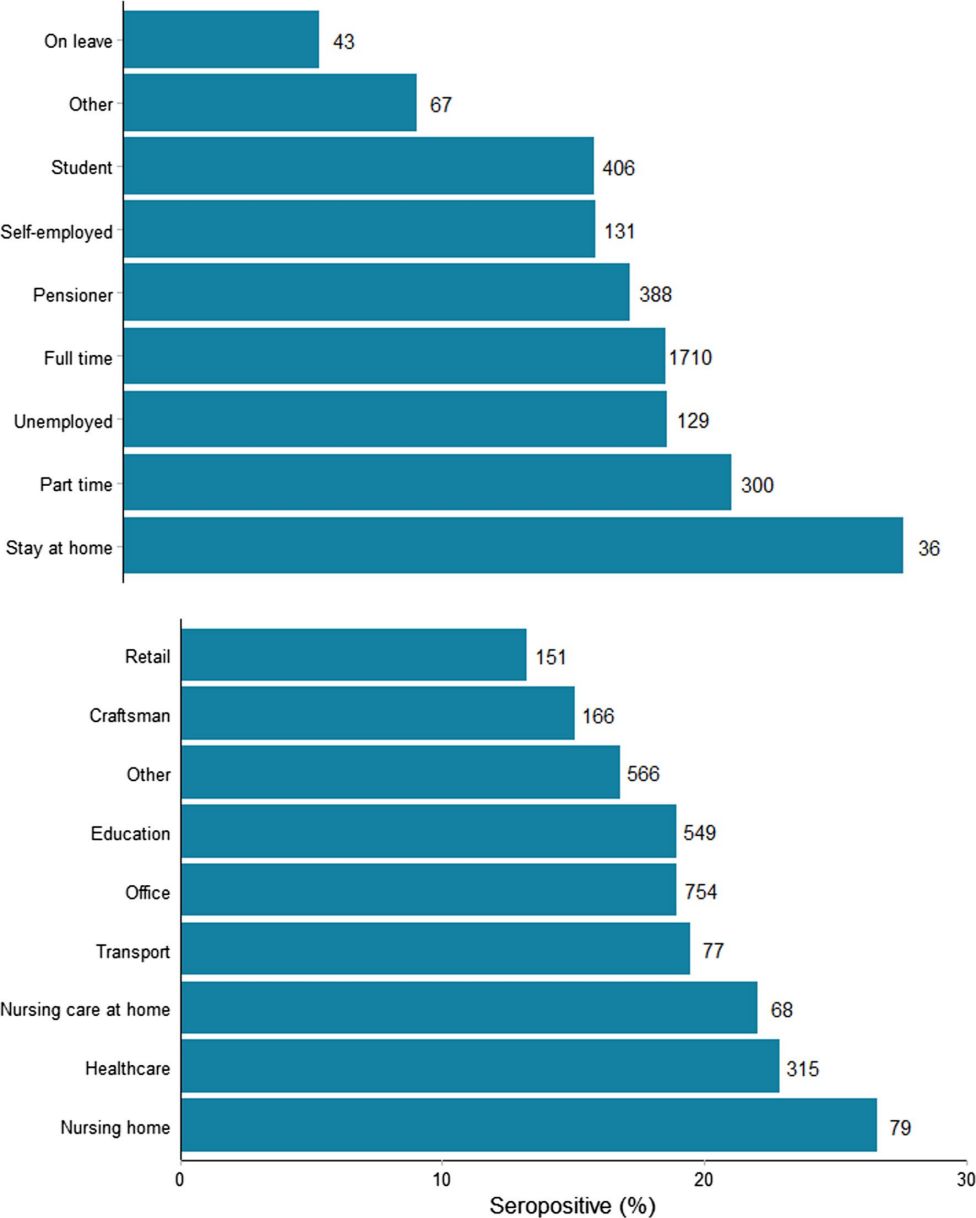
SARS-CoV-2 seroprevalence among 3210 individuals in SH areas by employment from questionnaire cohort

Of the 3236 participants who completed the questionnaire, 2141 (66.2%) reported working (part time, full time and self-employed participants combined). Figure 4 shows the risk of seropositivity stratified by employment status and occupation. Though a bit higher (19.7% vs 17.0%), in the adjusted model there was a 20% borderline significant increase in seropositivity among participants who reported working (OR 1.2, 95% CI 1.0–1.4, p = 0.06).

Seropositivity was higher in participants working at a nursing homes (n = 79, 22.9%), participants working in home care nursing (n = 68, 22.9%) and participants working in health care (n = 315, 22.1%). Compared to those with other occupations, working in health care or at nursing home was associated with a 30% higher risk of seropositivity (OR 1.3, 95% CI 1.0–1.6, p = 0.05).

Among the participants who completed the questionnaire, symptoms were reported by 2355 (72.8%). We found a significant association between reporting of symptoms associated with COVID-19 and seropositivity (RR 1.5, 95% CI 1.3–1.9, p < 0.001). Additional file 1: Figure S2 shows the frequency of each symptom stratified for seropositivity. Anosmia (loss of sense of smell) (RR 3.2, 95% CI 2.8–3.7, p < 0.001) and ageusia (loss of sense of taste) (RR 3.3, 95% CI 2.9–3.8, p < 0.001) had the strongest correlation to seropositivity. Additional file 1: Figure S3 is a forest plot of risk of seropositivity for each symptom.

### Adherence to the general COVID-19 recommendations from the Danish authorities

The national COVID-19 health recommendations are listed in Additional file 1: Figure S4. We found that only 53 (1.6%) participants reported not following any of the national COVID-19 recommendations, listed in the questionnaire (see Additional file 1: Appendix). Of those 41 were seronegative (77.4%) and 12 seropositive (22.6%), this was not significantly different from other participants (p = 0.48). Changes in behavior in response to the COVID-19 epidemic are shown in Additional file 1: Figures S5, S6. We found that the younger participants were less likely to follow general recommendations. There was no significant association between any behavioral change and seropositivity.

## Discussion

In this large, national, cross-sectional study we determined the seroprevalence of SARS-CoV-2 antibodies in SH areas. The findings from our study indicate that the prevalence of seropositive participants living in SH areas in Denmark was three times higher than in the general population. Males were found to be seropositive more often than females, similar to what has been reported in other studies [12, 13]. We found that seroprevalence increased with age, especially among men. Seroprevalence varied between geographical areas, being highest in the Capital region. The prevalence of self-reported symptoms in our study is consistent with previous findings with anosmia and ageusia having the strongest correlation to seropositivity [13, 14]. Regarding behavioral factors we saw that seropositive participants were less likely to smoke, drink alcohol or use drugs. This may be explained by a higher risk of seropositivity in older people, while alcohol intake, drug use and smoking are expected to be more widespread among young people, who generally had a lower risk of being seropositive. Another likely contributing explanation might be that people with such risk factors did not participate in the study. We found no significant difference in body-mass index (BMI) between seronegative and seropositive participants, however overall participants were overweight with a median BMI above 25.

In relation to age-related seroprevalence we do not see the same trend as in other Danish studies. The Danish National Seroprevalence Survey of SARS-CoV-2 infection by SSI described seroprevalence estimates were roughly 3 times higher in those aged 12–29 compared to 65 years and above [14] also, a study of 29,295 health-care workers in Denmark found participants under 30 years of age having the highest seroprevalence [13]. A study of 1100 retired blood donors found a lower seroprevalence in the age group ≥ 70 years [22], this was also seen in a study of ambulance staff in Sweden and Denmark which described the lowest proportion of seropositive over age 60 years [23].

Appropriate quarantine and separation from infected household members can be challenging in large households, and previous studies described how crowded living conditions constitute an increased risk of seropositivity [7, 8, 24, 25]. Furthermore, crowded living conditions is considered a key reason why people of low socioeconomic status or of ethnic minority backgrounds in particular have been disproportionately affected by the pandemic [4, 26]. Our results show that being exposed to SARS-CoV-2 due to close contact (physical contact and staying in the same room above 15 min as infected) or having someone infected in the household increased the risk of seropositivity significantly.

We found that the household composition was of importance, as living at least four people in a household or living in a multigenerational household increased the risk of seropositivity among participants. This finding is consistent with a previous preprint study on SARS-CoV-2 transmission within Danish households, which demonstrated an increased transmission risk with age [27].

Joblessness and low levels of education are more common in SH areas than in the general population and a study from Italy reported that being less educated may be a challenge under a pandemic, as the language barrier may affect the adherence to institutional recommendations such as wearing protective masks, avoid contagion and maintaining social distancing [5]. In our study only 2.7% seropositive participants answering the questionnaire were without any formal education. Our findings did not indicate clear association between seropositivity and behavioral change overall, and we observed a high rate of behavioral change, especially among the elderly. Only 1.6% of participants indicated not having changed behavior in response to the COVID-19 pandemic, why study participants overall were likely to implement public health measures. Joblessness (stay at home, unemployed or on leave) was not associated with the risk of seropositivity. Joblessness can potentially be a protective factor against COVID-19 infection as more prone to stay at home and avoiding public transportation or meeting people outside the household [5].

Employment in health care or nursing (nursing homes and nursing care at home) was associated with a 20% higher seropositivity rate, however this was borderline significantly (p = 0.05). This can be due to the fact that these job functions have been less affected by restrictions of working from home and avoiding person-to person contact and thereby pose an increased risk of infection. Also, vaccination of health care workers and workers in nursing homes was introduced December 27th, 2020 by the Danish Health Authority and the Ministry of Health as part of the Danish vaccination program, why there is a small likelihood that these participants have been vaccinated and thereby present a positive POCT based on antibodies from the vaccine instead of naturally immunization.

### Ethnicity

A report from the SSI about ethnicity and SARS-CoV-2 infection showed that citizens of non-Western descent were overrepresented by a factor of three in relation to the part they constitute of the Danish population, which may be due to living conditions or employment with higher risk of infection [8]. Unfortunately, we could not explore differences by ethnicity, as this information was not available by questionnaire and only a fraction of the participant informed about their social security number with the possibility for obtain information about ethnicity. In future studies like this it will be beneficial to include ethnicity in the questionnaire and include a translator at the test site to prevent potential linguistic barriers in answering of the questionnaires. Selection bias due to language barriers might have affected our results despite the fact that information material and questionnaire were available in seven different languages.

### Strengths and limitations

Our study has several limitations. The study design does not provide information on the point of time when participants became seropositive nor determination of time of infection. It is possible that participants who tested positive for SARS-CoV-2 earlier (viral throat- or nasopharyngeal swab or antibody test) or experienced COVID-19 like symptoms did not participate, leading to an underestimation of the true seroprevalence or the opposite, that those who knew they had been ill wanted to know if they had antibodies, and thereby an potential overestimation of the true seroprevalence. Seropositivity can be underestimated due to the fact that antibodies in response to SARS-CoV-2 infection can first be detected about 1 week after symptom onset [24, 28]. Furthermore, using seropositivity of Danish blood donors in the same period as a proxy for the general population is with some limitations to consider. Blood donors were in the age group 17–70 years and our study group are in the age group 15–83 years. Another source of error is that seroprevalence in blood donors is determined on the basis of ELISA, while the participants were tested with a POCT. In general, blood donors are in good health, why seropositivity in this group could be lower than expected. There is a tendency for health care professionals to be overrepresented as blood donors, and this group is found to have a higher risk of SARS-CoV-2 infection [13], why the seropositivity of blood donors could be higher than expected. A further caveat is that participants did not have to document a residential address in the SH areas, meaning that there may be participants included from other areas or from the same household, which could result in the seroprevalence being influenced by household clustering, thereby overestimating seroprevalence, especially in SH areas with few participants. There is a low risk of participants from SH areas having been vaccinated prior to participation in this study and thereby seropositive based on vaccine response and not natural immunization. Vaccinated blood donors have been removed from data.

This study had a broad national participation, which yields a representative sample of the Danish population living in SH areas of low socioeconomic status. The high participation rates across the country may reflects a keen interest in knowing the serological status supported by easily accessible testing facilities near the household, and written information in different language. Interobserver variation was limited as the POCT was read by project staff at the test sites. Serological surveys are the best tool to determine the spread of an infectious disease, particularly in the presence of asymptomatic individuals or incomplete ascertainment of those with symptoms. The POCT is a useful serological tool as it is easy to use, provides results in 15 min, can be performed by the participants, do not require a venous blood sample nor laboratory equipment and is less costly than ELISA, and thereby a suitable option for large sero-epidemiological studies.

## Conclusions

People living in SH areas in Denmark, have a three times higher seroprevalence of SARS-CoV-2 antibodies compared to the general Danish population. Seroprevalence was significantly higher for males and increased with age. Living in multiple generations households or in households of more than four persons was a strong risk factor for being seropositive. Results of this study can be used for future consideration of the need for preventive measures in the populations living in SH areas.

## Supplementary Information

**Additional file 1: Figure S1.** Map of seropositivity (made with Rstudio, own source). **Figure S2.** Frequency of symptoms among 3236 individuals in SH areas stratified by seropositivity. **Figure S3.** Forest plot of risk ratios (RR) for each symptom reported by questionnaire cohort. **Figure S4.** General recommendations from the Danish Health authorities. **Figure S5.** Change of behavior among 3236 individuals in SH areas during the pandemic stratified by seropositivity. **Figure S6.** Change of behavior among 3,236 individuals in SH areas during the pandemic stratified by sex and age in quartiles.

**Additional file 2: Table S1.** Seroprevalence of SH areas compared to their surrounding municipality.

## Data Availability

The datasets used and/or analyzed during the current study are available from corresponding author on reasonable request. The data are not publicly available due to Danish legislations.

## Abbreviations

SH: Social housing
POCT: Point-of care test
