# Supplementary material for: Seroprevalence of SARS-CoV-2 antibodies in social housing areas in Denmark
Source: BMC Infect Dis. 2022 Feb 10;22:143. doi: 10.1186/s12879-022-07102-1 (PMC8830972; doi:10.1186/s12879-022-07102-1)
Supplement: Supplementary file 1 — Additional file 1: Figure S1. Map of seropositivity (made with Rstudio, own source). Figure S2. Frequency of symptoms among 3236 individuals in SH areas stratified by seropositivity. Figure S3. Forest plot of risk ratios (RR) for each symptom reported by questionnaire cohort. Figure S4. General recommendations from the Danish Health authorities. Figure S5. Change of behavior among 3236 individuals in SH areas during the pandemic stratified by seropositivity. Figure S6. Change of behavior among 3,236 individuals in SH areas during the pandemic stratified by sex and age in quartiles. [file 12879_2022_7102_MOESM1_ESM.docx]

**Figure S1:** Map of seropositivity (made with Rstudio, own source)

##
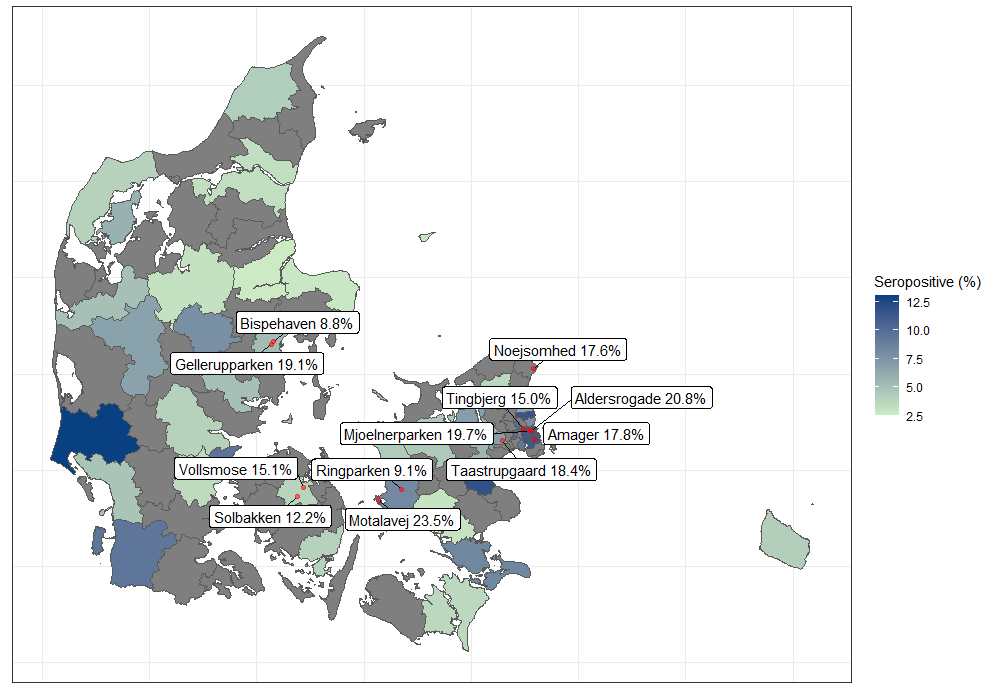


*Colors indicating the general population (blood donors)*

*Text boxes indicating the study cohort.*

*Gellerupparken: Including SH area Gellerupparken and Bazar Vest*

*Noejsomhed: Including SH area Noejsomhed and SH area Vapnagaard*

**Figure S2**: Frequency of symptoms among 3,236 individuals in SH areas stratified by seropositivity.


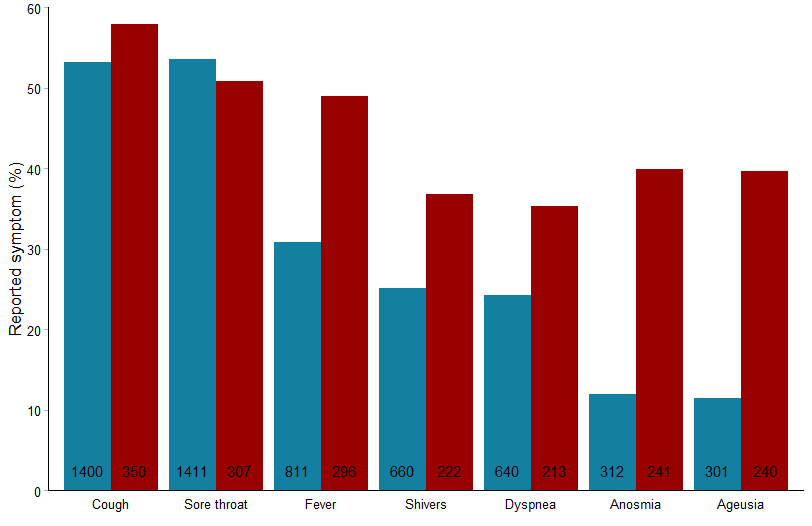


*Red: Seropositive, Blue: Seronegative*

**Figure S3**: Forest plot of risk ratios (RR) for each symptom reported by questionnaire cohort.

##
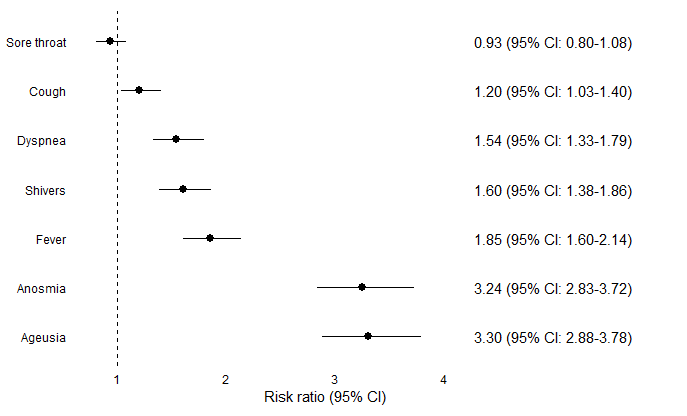


**Figure S4**: General recommendations from the Danish Health authorities

| 1 | Keep a distance of 2 meters whenever possible and always at least 1 meter |
| --- | --- |
| 2 | Avoid handshakes, hugs and kisses on the cheek |
| 3 | Self-isolate and take a PCR test if you have symptoms of COVID-19 |
| 4 | Open windows and doors and ventilate your home regularly |
| 5 | Avoid being too many people together indoors |
| 6 | Cough or sneeze into your sleeve |
| 7 | Wash your hands often or use hand sanitizer |
| 8 | Clean thoroughly and regularly, especially surfaces that are touched by many people |
| 9 | Persons aged 12 or more must wear face masks or shields in following locations: public transportation, wholesale and retail shops, at premises for cultural, sports and club activities, religious communities, day-care facilities, schools, education institutions, public and private hospitals and clinics and premises where clients are offered services involving physical contacts |

**Figure S5**: Change of behavior among 3,236 individuals in Danish SH areas during the pandemic by seropositivity


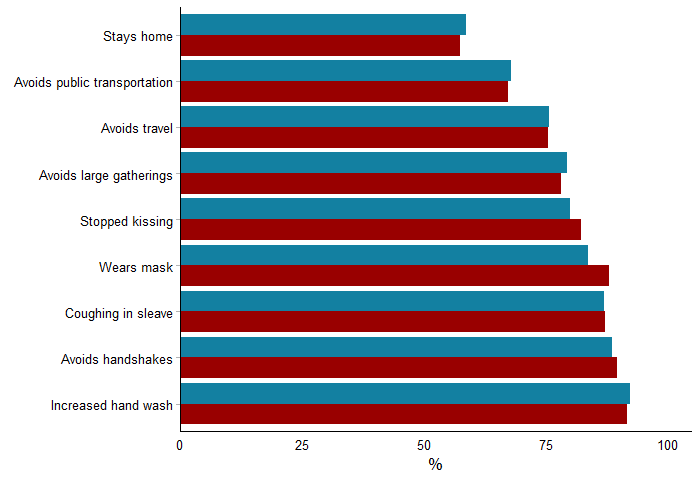


*Red: seropositive, Blue: seronegative*

**Figure S6**: Change of behavior among 2,871 individuals in SH areas by sex and age in quartiles


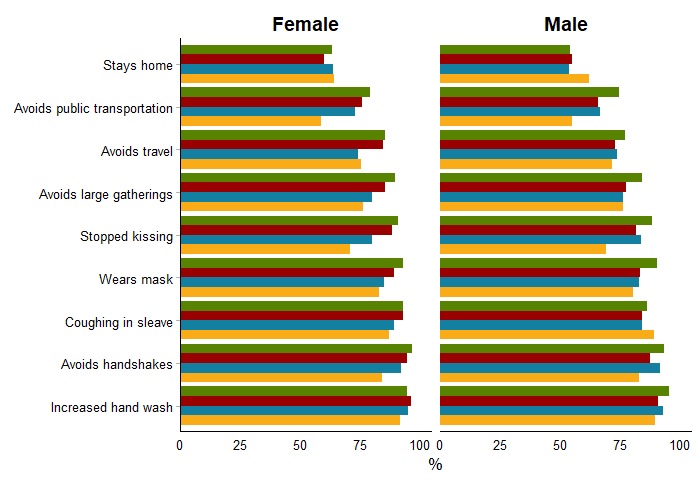


*(Green – age >56 years, red – age 45-56 years, blue – age 31-45 years, yellow - <31 years)*

**Appendix**

**List of test sites:**

| Copenhagen | Mjoelnerparken |
| --- | --- |
|  | Tingbjerg |
|  | Aldersrogade |
|  | Amager |
|  | Taastrupgård |
| Helsingoer | Noejsomhed |
|  | Vapnagaard |
| Slagelse | Ringparken |
| Korsoer | Motalavej |
| Aarhus | Bispehaven |
|  | Gellerupparken and Bazar Vest |
| Odense | Solbakken |
|  | Vollsmose |

**The questionnaire:**

| **Introductory questions** | |
| --- | --- |
| Do you want to state your civil registration (CPR) number? | 🌕 Yes  🌕 No |
| What is your civil registration number (without hyphen)? | _________________________ |
| Confirm your civil registration number (without hyphen) | _________________________ |
| THE CIVIL REGISTRATION NUMBERS DO NOT MATCH. PLEASE CHECK ABOVE AND ENTER AGAIN (This field disappears when the civil registration numbers entered in the above fields are identical) | _________________________ |
| What is your gender? | 🌕 Male  🌕 Female  🌕 Other  🌕 Don’t wish to answer |
| What is your date of birth? | _________________________ |
| What is your postcode? | _________________________ |
| How much do you weigh in kilograms (kg)? | _________________________ |
| How tall are you in centimetres (cm)? | _________________________ |
| Do you wish to do a finger prick test for COVID-19? | 🌕 Yes  🌕 No  🌕 Don’t know |
| May we compare the results of this survey with public registers? | 🌕 Yes  🌕 No |

| **Questions about your antibody tests**  **This part of the questionnaire concerns the antibody tests you have done in connection with We are testing Denmark** | | | | | | |  |
| --- | --- | --- | --- | --- | --- | --- | --- |
| What is the result of your finger prick test? | | | 🌕 Negative  🌕 Positive for IgM  🌕 Positive for IgG  🌕 Positive for both IgM and IgG  🌕 Inconclusive  🌕 I have not done the test/the test failed | | | |  |
| **Questions about COVID-19 infection**  **This part of the questionnaire concerns whether you have been infected with COVID-19.** | | | | | | |  |
| If you were to give your best assessment, do you think/know that you have ever been infected with COVID-19? | | | 🌕 Yes, I know/think that I have had COVID-19  🌕 No, I know that I haven’t had/I don’t think that I have had COVID-19 | | | |  |
| Have you previously been tested for COVID-19? | | | 🌕 Yes  🌕 No | | | |  |
| What type of COVID-19 test have you taken?  Select one or both answer options | | | □ Swab (sample from throat, nose or pharynx)  □ Antibody test (finger prick test or blood test)  □ Don’t know/don’t wish to answer | | | |  |
| Was one or more of your tests positive? | | | 🌕 Yes  🌕 No | | | |  |
| **Symptoms**  **The next questions concern your general health and whether you have had symptoms of COVID-19.**  **Have you had one or more of the following symptoms since 1 February?**  **(Select an answer for each option)** | | | | | | | |
|  | No | Yes, slight | | Yes, some | Yes, pronounced | Don’t know | |
| Fever | 🌕 | 🌕 | | 🌕 | 🌕 | 🌕 | |
| Chills | 🌕 | 🌕 | | 🌕 | 🌕 | 🌕 | |
| Impaired sense of smell | 🌕 | 🌕 | | 🌕 | 🌕 | 🌕 | |
| Impaired sense of taste | 🌕 | 🌕 | | 🌕 | 🌕 | 🌕 | |
| Sore throat | 🌕 | 🌕 | | 🌕 | 🌕 | 🌕 | |
| Cough | 🌕 | 🌕 | | 🌕 | 🌕 | 🌕 | |
| Difficulty breathing/shortness of breath | 🌕 | 🌕 | | 🌕 | 🌕 | 🌕 | |

| What do you think is/was the cause of your symptoms?  Select one or more of the following answer options: | | | □ COVID-19  □ Influenza or an influenza-like disease  □ Common cold  □ Allergy/hay fever  □ Asthma  □ Gastrointestinal infection  □ Other  □ Don’t know | | |
| --- | --- | --- | --- | --- | --- |
| Which of the following conditions best describes how you felt when you were feeling the worst, while you had/suspected you had COVID-19? | | | 🌕 I had no symptoms  🌕 I was at home with symptoms, but felt well  🌕 I was bed-ridden at home with symptoms  🌕 I was admitted to hospital  🌕 I was admitted and on a ventilator. | | |
| **Risk of COVID-19**  **The following questions concern how great the risk is that you have been exposed to coronavirus.** | | | | | |
|  | Yes | No | | Don’t know | Not relevant |
| Have you stayed for minimum 15 minutes in the same room as an infected person? | 🌕 | 🌕 | | 🌕 | 🌕 |
| Have you had body contact with a person infected with COVID-19? | 🌕 | 🌕 | | 🌕 | 🌕 |
| Have you worked/studied with someone who was infected with COVID-19? | 🌕 | 🌕 | | 🌕 | 🌕 |
| Has someone in your household been infected with COVID-19? | 🌕 | 🌕 | | 🌕 | 🌕 |
| Has someone in your family or a friend outside your household been infected with COVID-19? | 🌕 | 🌕 | | 🌕 | 🌕 |
| **This part concerns behaviour** | | | | | |
| Have you taken any of the following measures due to the risk of COVID-19 infection?  Select all relevant answers: | | | □ I wash hands more often  □ I cough or sneeze into my sleeve  □ I use disposable tissues  □ I wear disposable face masks  □ I avoid shaking hands  □ I avoid greeting persons by hugging and/or kissing them on both cheeks  □ I limit my use of public transport  □ I avoid places where many people are gathered  □ I stay at home  □ I work more from home  □ I avoid travelling outside my own country and/or region  □ None of the above | | |
| Do you smoke? | | | 🌕 No  🌕 Yes – occasionally  🌕 Yes – daily, less than 10 times a day  🌕 Yes – daily, 10 or more times a day  🌕 Previously  🌕 Don’t wish to answer | | |

| Have you consumed alcohol in the past 12 months? | 🌕 Yes  🌕 No  🌕 Don’t know/don’t wish to answer |
| --- | --- |
| On how many days a week do you drink alcohol? | 🌕 0-1 days  🌕 2 days  🌕 3 days  🌕 4 days  🌕 5 days  🌕 6 days  🌕 7 days |
| How many units do you typically drink per week?  1 unit = 1 regular beer or 1 glass of red/white wine or 1 alcoholic soft drink or 1 alcoholic cider or 1 glass of fortified wine or 1 drink/cocktail or 1 acquavit/shot | _________________________ |
| Do you use euphoriant drugs? | □ No  □ Yes – occasionally  □ Yes – often  □ Previously  □ Don’t wish to answer |
| Did you get an influenza vaccine last autumn/winter 2019?  Select one of the following answer options: | 🌕 Yes  🌕 No  🌕 I don’t know/don’t remember |
| Did you get an influenza vaccine this autumn/winter 2020?  Select one of the following answer options: | 🌕 Yes  🌕 No  🌕 I don’t know/don’t remember |
| **Work and education**  **The following questions are about your education and your work** | |
| What is your highest level of completed education?  Select one of the following answer options: | 🌕 I have no formal education  🌕 Primary education  🌕 Secondary education (youth education)  🌕 Vocational training or short-term/medium-term higher education  🌕 Long-term higher education  🌕 Don’t know |
| What is your main occupation?  Select one of the following answer options: | 🌕 Full-time work  🌕 Part-time work  🌕 Self-employed  🌕 Student  🌕 Homemaker  🌕 Unemployed  🌕 On long-term sick leave or parental leave  🌕 Pensioner  🌕 Other |

| Which area(s) or type(s) of work best describe(s) your work?  Select one or more answers | | | | | □ Healthcare sector  □ Nursing home  □ Home care  □ Shop work  □ Office work  □ School/other educational establishment  □ Tradesman  □ Transport (Bus, taxi, train or other means)  □ Other  □ Don’t wish to answer | | | | |
| --- | --- | --- | --- | --- | --- | --- | --- | --- | --- |
| **The following questions concern your household**  **Information about your household is important to enable us to assess your risk of being infected with coronavirus and getting COVID-19.**  **How many people in the following age groups, including yourself, live in your household?**  **Please select one answer for each element.** | | | | | | | | | |
|  | None | 1 | 2 | 3 | | 4 | 5 | >5 | Not relevant |
| 0-4-year-olds | 🌕 | 🌕 | 🌕 | 🌕 | | 🌕 | 🌕 | 🌕 | 🌕 |
| 5-18-year-olds | 🌕 | 🌕 | 🌕 | 🌕 | | 🌕 | 🌕 | 🌕 | 🌕 |
| 19-44-year-olds | 🌕 | 🌕 | 🌕 | 🌕 | | 🌕 | 🌕 | 🌕 | 🌕 |
| 45-64-year-olds | 🌕 | 🌕 | 🌕 | 🌕 | | 🌕 | 🌕 | 🌕 | 🌕 |
| 65+-year-olds | 🌕 | 🌕 | 🌕 | 🌕 | | 🌕 | 🌕 | 🌕 | 🌕 |
| May we contact you later if we have elaborating further questions? | | | | | 🌕 Yes  🌕 No | | | | |
| What is your email address? | | | | | _________________________ | | | | |
| Confirm your email address | | | | | _________________________ | | | | |
| THE EMAIL ADDRESSES DO NOT MATCH. PLEASE CHECK ABOVE AND ENTER AGAIN (This field disappears when the email addresses entered in the above fields are identical) | | | | | _________________________ | | | | |
