# Supplementary material for: Seroprevalence of SARS-CoV-2 antibodies in social housing areas in Denmark
Source: BMC Infect Dis. 2022 Feb 10;22:143. doi: 10.1186/s12879-022-07102-1 (PMC8830972; doi:10.1186/s12879-022-07102-1)
Supplement: Supplementary file 2 — Additional file 2: Table S1. Seroprevalence of SH areas compared to their surrounding municipality. [file 12879_2022_7102_MOESM2_ESM.docx]

**Table S1:** Seroprevalence of SH areas compared to their surrounding municipality.

| **Municipality** | **SH area** | **Seroprevalence**  **In SH area** | **Seroprevalence**  **In municipality** |
| --- | --- | --- | --- |
| **Copenhagen** | Mjoelnerparken  Tingbjerg  Aldersrogade  Amager | 19.7  15.0  20.8  17.8 | 11.3 |
| **Hoeje-Taastrup** | Taastrupgaard | 18.4 | 6.8 |
| **Helsingoer** | Noejsomhed | 17.6 | UK |
| **Slagelse** | Ringparken  Motalavej | 9.1  23.5 | 8.2 |
| **Aarhus** | Bispehaven  Gellerupparken | 8.8  19.1 | 5.1 |
| **Odense** | Solbakken  Vollsmose | 12.2  15.1 | 3.8 |

*UK: Unknown according to the background population in that area*
